# Immunofluorescence Analysis of Duck plague virus gE protein on DPV-infected ducks

**DOI:** 10.1186/1743-422X-8-19

**Published:** 2011-01-15

**Authors:** Hua Chang, Anchun Cheng, Mingshu Wang, Renyong Jia, Dekang Zhu, Qihui Luo, Zhenli Chen, Yi Zhou, Fei Liu, Xiaoyue Chen

**Affiliations:** 1 Avian Diseases Research Center, College of Veterinary Medicine of Sichuan Agricultural University, Yaan, Sichuan, 625014, China; 2 Key Laboratory of Animal Diseases and Human Health of Sichuan Province, Yaan, Sichuan, 625014, China; 3 Epizootic Diseases Institute of Sichuan Agricultural University, Yaan, Sichuan, 625014, China

## Abstract

**Background:**

In previous studies, the expression and localization characteristics of duck plague virus (DPV) gE protein have been described in cultured cells, but the properties of DPV gE protein have not been reported in vivo. Immunofluorescence analysis had been used for the detection of virus antigen, but there was no report on the use of this technique for the detection of DPV gE. In this study, we investigated the distribution of DPV gE protein on DPV-infected ducks using polyclonal antibody raised against the recombinant His-gE fusion protein by indirect immunofluorescence assay (IFA).

**Results:**

The recombinant gE protein was highly immunogenicity by ELISA, and the gE was used as an antigen for the preparation of polyclonal antibody, which could be used the first antibody for further experiment to study the distribution of DPV gE protein in DPV-infected tissues by indirect immunofluorescence assay. DPV gE protein were distributed in the immune organs (thymus, bursa of fabricius (BF), Harders glands, spleen), the digestive organs (liver, duodenum, jejunum, ileum), and the other parenchymatous organs (kidney, myocardium, cerebrum, and lung) of DPV-infected ducks, but the positive immunofluorescence signal was not seen in the muscle and pancreas. The lymphocytes, reticulum cells, macrophages, epithelial cells, and hepatocytes served as the principal site for the localization of DPV gE antigen. Moreover, the intensity of fluorescence increased sharply from 12 to 216 h post-infection (p.i.).

**Conclusions:**

In this work, the immunogenicity of the recombinant gE protein was analyzed by ELISA, and we presented the distribution properties of DPV gE antigen in infected ducks for the first time, which may be useful for understanding the pathogenesis of DPV. These properties of the gE protein provided the prerequisite for further functional analysis.

## Background

Duck plague (DP) is an acute contagious disease that is highly lethal in all ages of birds from the order Anseriforms (ducks, geese, and swans) [1]. The characterization of duck plague is tissue hemorrhage, digestive mucosal eruptions lesions of lymphoid organs and degenerative changes in parenchymatous organs [2]. Duck plague was difficult to monitor and control, because duck plague virus established an asymptomatic carrier state in both domestic and wild waterfowls that was detectable only during the intermittent shedding period of the virus [3]. Duck plague has resulted in significant economic losses in commercial duck industry due to high mortality rate and decreased duck egg production [4].

Glycoprotein E (gE) encoded by US8 from Alphaherpesvirinae had demonstrable effects on virulence and spread in the nervous system, and played important roles in determining the extents of cell-to-cell spread, perhaps by binding a ligand while on the surface of an infected cell and signaling through its cytoplasmic sequences to affect gene expression in the infected cells [5,6]. The duck plague virus (DPV) gE protein is a 490-amino acid glycoprotein protein encoded by US8 gene. At present, some studies showed immunofluorescence assay (IFA) method had been widely used for the detection of specific pathogen, virus, and bacteria [7,8], but no report was available on the use of this technique for the detection of duck plague virus (DPV) gE protein. In this study, using polyclonal antibody raised against the recombinant His-gE fusion protein, the distribution of DPV gE was investigated in paraffin-embedded tissues of experimentally DPV-infected ducks by indirect immunofluorescent staining method.

## Results

### Expression and Immunogenicity of DPV gE protein

**Figure 1:**
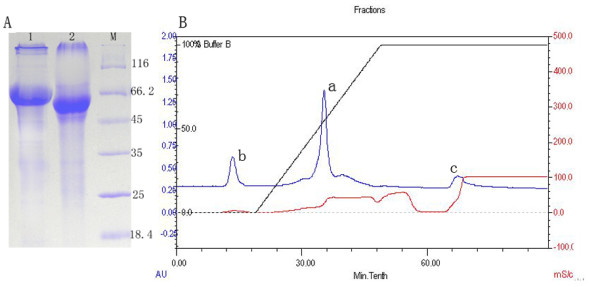
**SDS-PAGE of the purified gE protein and the purified serum**. A. SDS-PAGE of the purified gE protein and the purified serum. Lane 1, the purified gE protein; Lane 2, the purified serum; Lane M, protein marker. B. The purified serum was collected at a. The b and c were faint impure peaks.

**Figure 2:**
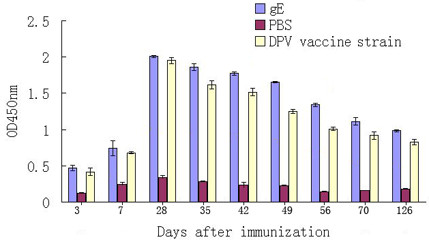
**The immunogenicity of gE protein by ELISA**. Purified gE proein was coated and the sera from immunized ducks were used as primary antibody. At 28 days, the OD450 nm values reached maximum value.

DPV gE protein was overexpressed in E.coil Rosetta, and purified as an antigen for antibody development (Figure 1.A). The result of ELISA indicated that the recombinant protein was observed to be highly immunogenic. On 7 days, the OD450 nm value obtained was 0.702, while unimmunized ducks showed the OD450 nm value of 0.247, and the OD 450 nm value of immunized ducklings with DPV commercial attenuated vaccine strain was 0.681. At 28 days, the OD450 nm values reached maximum value (2.009) after inoculation, and the antibody titers of DPV gE protein continued to have a high level for 126 days (Figure 2).

### The purification of the His-gE antiserum and Optimum conditions of IFA

The rabbit polyclonal antiserum raised against the recombinant gE protein were prepared, and the His-gE antiserum was purified, the IgG was collected (Figure 1.B), and examined by SDS-PAGE (Figure 1. A). The purified gE antiserum was subsequently used as primary antibody in indirect immunofluorescent staining method. The optimum conditions of IFA for DPV gE antigen detection were as follows: Endogenous peroxidase activity was blocked by 0.3% hydrogen peroxide (H_2_O_2_) in methanol for 45 min, antigen recovery was performed on microwave with citrate buffer solution (0.01 M, PH 6.0) for 20 min, the sections were incubated with 10% normal goat serum for 2 h at 37°C, and incubated the primary antibody (1:100) for 60 min at 37°C. The secondary antibody was diluted 1:150, and incubated for 45 min at 37°C.

### Detection of DPV gE antigen

Following experimental DPV infection, the tissues were obtained from the infection group and the controls. The systemic distribution of DPV gE antigen by IFA was summarized in Table 1. The first positive fluorescent signals of DPV gE were seen in the BF and spleen at 4 h post-infection (p.i.). Then the positive signals were detected Harderian gland, thymus, liver, duodenum, jejunum, and ileum at 8 h p.i., and a number of positive signals were shown in the kidney, myocardium, lung, and cerebrum 12 h p.i., whereas no positive signals were detected in the muscle and pancreas. And the fluorescent intensity of positive signals increased with the infection times from 12 h to 216 h p.i..

**Table 1:** The immunofluorescence analysis of DPV gE antigen on DPV-infected ducks

Tissues		Hours post DPV infection (h)									
		
		2	4	8	12	24	72	96	120	168	216
	Spleen	-	+	+	++	+++	+++	+++	+++	+++	+++
Lymphoid organs	BF	-	+	+	++	++	+++	+++	+++	+++	+++
	Harderian gland	-	-	+	+	+	++	++	+++	+++	+++
	Thymus	-	-	+	+	++	++	+++	+++	+++	+++
	Liver	-	-	+	+	++	+++	+++	+++	+++	+++
Digestive organs	duodenum	-	-	+	+	++	+++	+++	+++	+++	+++
	jejunum	-	-	+	+	++	+++	+++	+++	+++	+++
	ileum	-	-	+	+	++	+++	+++	+++	+++	+++
	Kidney	-	-	-	+	++	+++	+++	+++	+++	+++
Other organs	Myocardium	-	-	-	+	+	++	++	++	+++	+++
	cerebrum	-	-	-	+	+	+	++	++	+++	+++
	Lung	-	-	-	+	+	++	++	+++	+++	+++

negative(-), no fluorescing cells; weakly positive(+), the signal was faintly cells (less than 5%); positive(++), 5% to 50% was readily detected,; strongly positive(+++), the positive staining is intense more than 50%.

**Figure 3:**
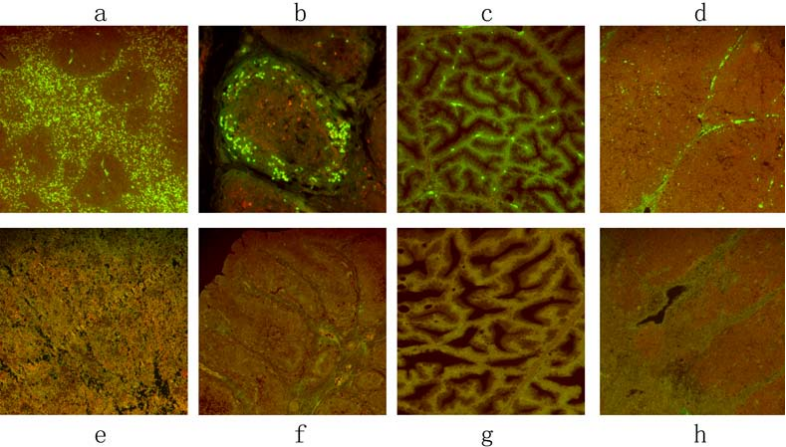
**The immunofluorescence distribution of DPV gE antigen on immunological organs**. The tissue sections were stained with indirect immunofluorescent assay at 24 h post-infection. The positive signal of the gE antigen appeared yellow-green, and the positive staining was widely distributed in a to d (spleen, BF, Harderian gland, thymus); and no positive signals were detected on the spleen, BF, Harderian gland, thymus of mock-infected ducks (e to h).

DPV gE antigen was intensely detected in the lymphoid organs. The positive fluorescent signals for DPV gE antigen were mostly found in the red pulp and white pulp of spleen (Figure 3a), in the follicles of the bursa of fabricius (Figure 3b), in the glandular acini of Harders glands (Figure 3c), and in the cortex and medulla of thymus (Figure 3d) at 24 h p.i.. The fluorescent signals were mostly located in the lymphocytes, reticulum cells, macrophages and epithelial cells.

**Figure 4:**
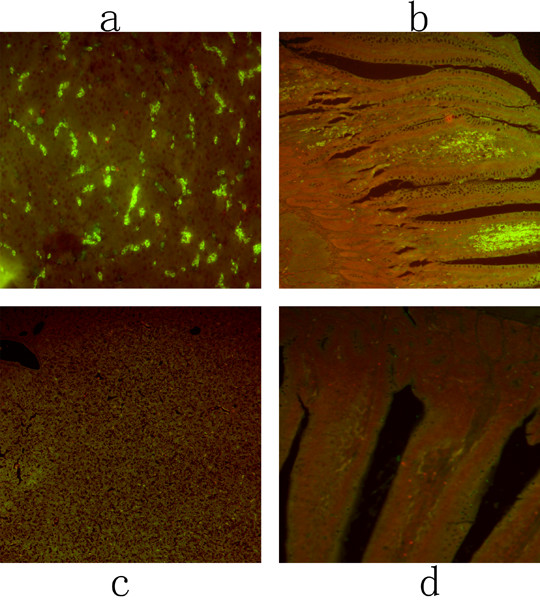
**The immunofluorescence distribution of DPV gE antigen on digestive organs**. The tissue sections were detected at 24 h post-infection. The positive signals were yellow-green, and widely distributed in a and b (liver and duodenum); and no positive signals were detected on the liver and duodenum of mock-infected ducks (c and d).

DPV gE antigen was widely detected in the digestive organs. The positive signals were mostly found in the hepatic lobules of liver (Figure 4a), the mucous membrane, intestinal glands and submucosa of the intestine (Figure 4b) at 24 h p.i.. And the fluorescent signals were detected in the hepatocytes of the hepatic lobules, and in the superficial mucosal cells, intestinal glandular cells, and fibrocytes of submucosa.

**Figure 5:**
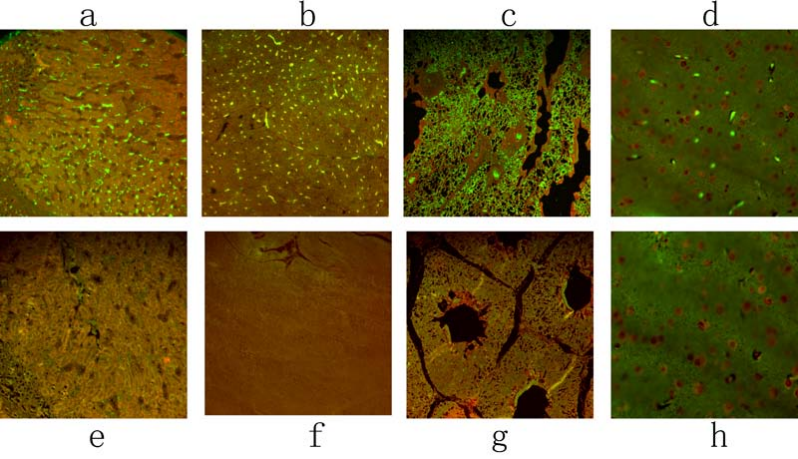
**The immunofluorescence distribution of DPV gE antigen on other parenchymatous organs**. The tissue sections were detected at 96 h post-infection. The positive signals were yellow-green, and intensely distributed in a and d (kidney, myocardium, lung, and cerebrum); and no positive signals were detected on the kidney, myocardium, lung, and cerebrum of mock-infected ducks (e to h).

In addition, the positive fluorescent signals for DPV gE antigen increased in the kidney, myocardium, lung, and cerebrum at 96 h p.i.. The positive signals were found in the epithelial cells of the renal tubule (Figure 5a), in the myocardium fibrocytes (Figure 5b), in the endothelial cells of the pulmonary alveolus (Figure 5c) and in the glia cells of cerebral cortex (Figure 5d).

### Specific detection

Specific fluorescent signal for DPV gE antigen was intensely found in immunological organs and digestive organs from the DPV-infected ducks, while no positive signal was located in the tissues of mock-infected ducks, and Duck hepatitis virus (DHV), Marek's disease virus (MDV), New type gosling viral enteritis virus (NGVEV)-infected birds during the whole experiment (Table 2).

**Table 2:** The immunofluorescence analysis of DPV gE antigen on DHV, MDV, NGVEV, mock -infected birds

Tissues	DHV	MDV	NGVEV	mock-infected ducks
Lymphoid organs	-	-	-	-
Digestive organs	-	-	-	-
Other organs	-	-	-	-

## Discussion

The duck plague virus (DPV) gE protein is a 490-amino acid glycoprotein protein encoded by US8 gene. The glycoprotein gE play an important role in determining the virulence of duck plague virus. In our study, DPV gE fusion protein, with a relative molecular mass of 74 kDa, was expressed in E. coli Rosetta. The recombinant protein was surveyed to be highly immunogenic by ELISA, and purified as an antigen for antibody preparation. Previous study indicated that the gE envelope glycoprotein is very significant immunodominant antigen for the development of new antibodies, and the rabbit anti-His-gE polyclonal antibody had high reactivity and specificity for DPV gE [9,10]. It demonstrated that the antiserum could be used as the primary antibody for detecting the distribution of gE protein on DPV-infected ducks.

IFA was a highly specific and sensitive diagnostic method for detecting infectious diseases [11-13]. In this report, the DPV gE antigen on DPV-infected ducks was examined by IFA. The result demonstrated that DPV gE antigen was extensively present in immunological organs and digestive organs. DPV gE antigen was detected firstly in Lymphoid organs (Bursa of Fabricius, thymus, spleen, and Harderian gland) (Figure 3.). The thymus and BF were the primary immune organs, where the differentiation and maturation of T lymphocytes and B lymphocytes generated, respectively [14,15]. Harderian gland and spleen were the secondary immune organs. There are many interstitial aggregations of B-dependent lymphoid cells in the Harderian gland [16-18], and spleen is an abundant source of immunocompetent cells (T and B lymphocytes) [19-21]. T and B lymphocytes played important roles in mediating immune responses. It could offer that the lymphoid organs were damaged seriously, after immune responses to heteroantigen in lymphoid organs of ducks infected with duck plague virus.

The immunofluorescence signal appeared in the liver and small intestine (including the duodenum, jejunum, and ileum) as early as 8 h p.i. (Figure 4.). These results indicated that the DPV-infected ducks occurred mucosal damage of digestive organs after the normal defence mechanism was damaged. These results of the present study were in accordance with previous studies. Shen had reported that the bursa of Fabricius, thymus, spleen, liver, and intestine were the main target organs of DPV in acute DPV cases [22]. Furthermore, the intensity of positive immunofluorescence signal of DPV gE antigen in various tissues increased sharply from 12 to 216 h p.i.. Duck plague virus proliferated quickly in lymphoid organs and the digestive organs, finally, the other parenchymatous organs (kidney, myocardium, lung and cerebrum) were invaded by blood circulation (Figure 4.). In addition, DPV gE could induce cell fusion and promote cell-to-cell spread of the virus in infection process [23,24] and the release of the virus [25,26], so that it was easy to accelerate the progress of the infection. And gE could promote the virus to transport the central nervous system in infection [27], which was damaged. These data also indicated that DPV would infect a variety of tissues in ducks, and additional replication in these sites caused a major viraemia.

## Conclusions

In conclusion, we described for the first time the basic characteristics of DPV gE distribution in the tissues of experimentally DPV-infected ducks by indirect immunofluorescence assay. From these results of immunofluorescence studies, it concluded that DPV gE mainly located in the immunological organs and digestive organs. This research will provide new insights into understanding the pathogenesis of DPV. Further studies will be aimed at constructing of DPV gE mutant to study the function of DPV gE.

## Materials and methods

### Virus strain, Immunogenicity Analysis of gE Protein, and Antibody preparation

DPV CHv strain was a high-virulence field strain, obtained from Avian Disease Research Centre of Sichuan Agricultural University. DPV commercial attenuated vaccine strain was provided by Key Laboratory of Animal Disease and Human Health of Sichuan Province. The gE fusion protein was expressed in Escherichia coli as our laboratory described previously [10,28,29]. His-gE recombinant protein was expressed in Escherichia coli Rosetta induced by isopropy1-β-D-thiogalactopyranoside (IPTG) at 30°C for 4.5 h. Over expressed His-gE fusion protein were purified using a Ni-NTA affinity chromatography.

And the immunogenicity analysis of His-gE protein was detected by ELISA as our laboratory described previously [30]. The procedures were as follows: 96-well plates were coated with the purified gE protein (1:100) overnight at 4°C and blocked with 1% BSA in PBST (PBS containing 0.05% Tween-20) for 1 h at 37°C. After three washings with PBST, the sera from immunizing ducklings (at 3, 7, 28, 35, 42, 49, 56, 70, 126 days after immunizing the gE protein) were diluted (1:160) to the wells and incubated for 1 h at 37°C. Finally, after washing three times, plates were incubated with HRP-conjugated goat anti-duck IgG (1:1000) (KPL, USA) for 1 h at 37°C. Then, the antibody was removed, and the plates were washed 3 times with PBST. The reaction was developed with the 3'3'5'5-tetramethylbenzidine (TMB) substrate with H_2_O_2_. The optical density was measured at 450 nm using Bio-Rad 860 reader (Bio-Rad, USA) after stopping the reaction with 2 mol/L H_2_SO_4_. The control groups were immunized with DPV commercial attenuated vaccine strain as positive group and phosphate-buffered saline solution (PBS) as negative group.

Then, the rabbit polyclonal antiserum raised against the recombinant gE protein were prepared as described previously [10]. And the antiserum was purified by ammonium sulfate precipitation and High-Q anion-exchange chromatography using BioLogic LP system [31]. At first, the column was washed in 100% solvent A (HPLC water) for 30 min. The column was equilibrated in 100% solvent A (20 mM Tris-HCl) for 20 min, then the sample (the antiserum) was loaded in the columm, and the columm was equilibrated in 100% solvent A (20 mM Tris-HCl) for 10 min. Finally, the column was eluted with linear gradient of 100%A (20 mM Tris-HCl)/0%B(1 M NaCl) to 0%A(20 mM Tris-HCl)/100%B(1 M NaCl) for 30 min and the sample was collected and analyzed by SDS-PAGE.

### Experimental animals and sampling

Thirty five 30-day old Cherry Valley ducks (not vaccinated against DPV) were divided randomly into two groups. Twenty-five ducks were intramuscularly inoculated with DPV CHv strain at a dose of 0.2 ml containing 10^3 ^MLD (minimum lethal dose). The remaining ducks were intramuscularly with 0.2 ml PBS (0.01 M, pH 7.4) instead of virus and used as the negative control. The ducks were housed in isolation units in a biosecure building and provided with a commercial duck diet ad libitum.

At 2, 4, 8, 12, 24, 72 h postinfection (p.i.), and later every 1 day p.i. until they died (216 h p.i.), two ducks were randomly taken from the infection group, and one duck was randomly selected in the controls and euthanatized at each time point. These tissues (Harderian gland, Bursa of Fabricus (BF), thymus, duodenum, jejunum, ileum, liver, spleen, pancreas, myocardium, lung, kidney, cerebrum, and muscle) were collected from DPV-infected ducks and fixed by 4% paraformaldehyde in 0.1 mol of phosphate buffer (PH 7.4). In addition, these tissue sections of duck hepatitis virus (DHV), Marek's disease virus (MDV), and new type gosling viral enteritis virus (NGVEV)-infected birds were provided by Key Laboratory of Animal Disease and Human Health of Sichuan Province.

### Optimization and application of Immunofluorescence assay (IFA)

Tissues were fixed, and processed for paraffin embedding, and cut at 4 um thickness. The sections were de-waxed in xylene and re-hydrated in PBS (0.01 M, PH 7.4) for 5 min. Endogenous peroxidase activity was blocked by immersing the slides in 0.3% hydrogen peroxide (H_2_O_2_) in methanol for 45 min (A) or 3% H_2_O_2 _in methanol for 15 min(B). Then the sections were submitted to antigen retrieval in represents microwave antigen retrieval in citrate buffer solution (CBS, 0.01 M, pH 6.0) for 20 min(A), 0.1% trypsase for 10 minutes at 37°C(B), or no recovery. After washing three times with (PBS containing 0.05% Tween-20, 0.01 M, PH 7.4), the slides were incubated with 10% normal goat serum or 10% bovine serum albumin for 2 h at 37°C. The blocking serum was tapped off, and the sections were covered with the purified rabbit-gE polyclonal antibody without dilution, at dilutions of 1:50, 1:100, or 1:200 in PBST containing 1%BSA, and incubated in a humidified chamber for 1 h at 37°C. After washing with PBST three times, the slides were covered with the fluorescein isothiocyanate (FITC)-labelled goat anti-rabbit IgG diluted 1:50, 1:100, 1:150, or 1:200 in PBST containing 1%BSA for 45 min, 60 min, or 90 min at 37°C. Finally, the sections were lightly counterstained with 0.01% Evans blue at room temperature, and examined through a fluorescent microscope (Nikon 80i).

### Specificity assessment

The tissues of PBS-infected ducks, and other virus (DHV, MDV, NGVEV) infected birds were detected by IFA, respectively.

### Assessment of IFA

The positive for gE antigen gave a yellow-green fluorescent signal (FITC staining), while those negative for gE antigen appeared red (Evans blue) by fluorescent microscope observation. The assessment of results was conducted as described previously, with some modifications [22,32,33], the percentage of positive cells were scored as follows: (a) no fluorescing cells, nagative(-); (b) less than 5% fluorescing cells, weakly positive(+); (c) 5% to 50% fluorescing cells, positive(++); (d) more than 50% fluorescing cells, strongly positive(+++).

## Competing interests

The authors declare that they have no competing interests.

## Authors' contributions

HC carried out most of the experiments and wrote the manuscript. ACC and MSW critically revised the manuscript and the experiment design. RYJ, DKZ, QHL, ZLC, YZ, FL, XYC helped with the experiment. All of the authors read and approved the final version of the manuscript.
